# Supplementation with Animal- and Plant-Derived Proteins Modulates the Structure and Predicted Metabolic Potential of the Gut Microbiota in Elite Football Players

**DOI:** 10.3390/nu18050768

**Published:** 2026-02-26

**Authors:** Bartosz Kroplewski, Katarzyna E. Przybyłowicz, Tomasz Sawicki, Sebastian Wojciech Przemieniecki

**Affiliations:** 1 Department of Human Nutrition, Faculty of Food Sciences, University of Warmia and Mazury in Olsztyn, ul. Słoneczna 45F, 10-718 Olsztyn, Poland; katarzyna.przybylowicz@uwm.edu.pl (K.E.P.); tomasz.sawicki@uwm.edu.pl (T.S.); 2 Department of Entomology, Phytopathology and Molecular Diagnostics, Faculty of Agriculture and Forestry, University of Warmia and Mazury in Olsztyn, Prawocheńskiego 17, 10-720 Olsztyn, Poland; sebastian.przemieniecki@uwm.edu.pl

**Keywords:** protein supplementation, whey protein, pea protein, rice protein, microbiome composition, football players, gut health, functional metagenomics

## Abstract

Background/Objectives: The primary outcome of this 8-week randomized, controlled, parallel trial was to assess longitudinal shifts in gut microbiota structure and predicted metabolic potential in 45 elite football players following protein supplementation. Methods: Participants combined resistance training with daily intake (30 g) of whey protein concentrate (WPC), pea protein isolate (PPI), rice protein isolate (RPI), or a plant-protein blend (MIX). For the acquisition of prokaryotic metataxonomic data, the V3–V8 region of the 16S rRNA gene was sequenced using Oxford Nanopore Technology (ONT). Functional potential was inferred through the MACADAM database and STAMP software. Strict dietary monitoring and gravimetric adherence checks were performed to isolate the intervention effect. Results: While microbial alpha-diversity indices (Chao1, Shannon, Simpson) remained stable across all groups, significant source-specific shifts in taxonomic structure and predicted metabolic activity were identified. Whey protein concentrate (WPC) was associated with an increase in *Bacteroidetes* abundance and greater balance within the microbial community structure, whereas pea protein isolate (PPI) and the MIX correlated with reduced fermentative bacteria and elevated taxa potentially involved in cadaverine biosynthesis. Rice protein isolate (RPI) supplementation was associated with a higher predicted representation of taxa involved in succinate-to-butyrate fermentation pathways. These functional markers and differential responses of selected bacterial groups to particular protein types were observed. Conclusions: The data indicate complex interactions between supplement type, exposure duration, and microbiome response, underscoring the necessity for individualized dietary recommendations and supplementation strategies to optimize gut health and training adaptation in professional football players.

## 1. Introduction

Recent years have seen increasing interest in the impact of diet on gut microbiota, especially among high-performance athletes. A major aspect is the role of protein supplements—both plant and animal-derived—in modulating microbiota composition. Understanding these relationships is crucial, as gut microbiota has many functions for overall health, exercise adaptation, post-exercise recovery, and athletic performance. The gut microbiota is essential for digestion, metabolism, vitamin synthesis, immune modulation, and maintenance of gut barrier integrity. Its composition and metabolic activity are influenced greatly by environmental factors, among which diet and supplementation are key [1,2,3].

Athletes often consume high-protein diets to increase muscle mass, improve recovery, and enhance strength. This macro-nutrient may have both beneficial and undesirable health effects regarding microbiota [4,5]. According to WHO, recommended daily protein intake for adults is 0.83 g/kg body weight, but athletes require much more—1.2 to 2 g/kg/day. Protein is essential for tissue repair, enzyme and hormone synthesis, and adaptation to training loads. Whey, casein, soy, and pea protein supplements are commonly used as convenient sources of high-quality protein. Many athletes opt for animal proteins due to their high biological value, complete amino acid profile, and rapid absorption. Plant proteins, sometimes considered less complete, are gaining popularity in vegan/vegetarian diets and for their potentially advantageous effects on metabolic health and the environment [6].

Digestibility and amino acid composition differences between protein types affect training outcomes and gut microbiota. About 10% of dietary protein escapes digestion in the small intestine and enters the large intestine, where it becomes a substrate for microbial fermentation. This produces various metabolites, including SCFAs (butyrate, propionate, acetate) with anti-inflammatory properties, serving as key energy sources for epithelial cells, and playing vital roles in host metabolism regulation. Fermentation also produces branched-chain fatty acids, ammonia, hydrogen sulfide, and phenolic and indole compounds, some of which may have toxic or pro-inflammatory effects. High protein diets may decrease SCFA production and increase potentially harmful metabolites, affecting gut barrier function, inflammation, and promoting dysbiosis [7].

Protein type and source are critical: whey and beef proteins promote different microbiota members than plant proteins like soy and pea. In a pilot study by Moreno-Pérez et al. [8], animal protein supplementation was compared to carbohydrate supplementation in endurance athletes. No significant changes in overall diversity, but marked alterations in certain taxa were noted, including increased *Bacteroidetes* linked to protein degradation, reduced *Firmicutes*, and lower levels of beneficial taxa (*Blautia*, *Roseburia*, *Bifidobacterium longum*). This decline in *B. longum* may impair gut barrier function and anti-inflammatory effects. Even short-term supplementation can cause rapid, measurable microbiota shifts affecting health and performance. Researchers lack adequate data on protein supplement effects on athlete gut microbiota—especially in elite cohorts such as football players, whose baseline microbiota and metabolic profiles may substantially differ, impacting responses to supplementation.

Therefore, our study aimed to evaluate the effects of animal- and plant-derived protein supplementation on the structure and metabolic potential of elite football players gut microbiota. Specifically, we sought to characterize microbiota profiles associated with different protein types and explore the potential for personalized supplementation strategies based on individual microbiome composition to support athletic performance and health. Understanding these relationships opens new avenues for optimizing nutritional approaches in athletes. The aim of the research was to assess changes in the gut microbiota structure and metabolic potential in football players undergoing supplementation with animal- and plant-derived proteins.

## 2. Materials and Methods

### 2.1. Study Design

This randomized, controlled parallel intervention was approved by the Bioethics Committee of the Collegium Medicum, University of Warmia and Mazury, Olsztyn, Resolution No. 11/2023, and registered in the ClinicalTrials.gov database (NCT06610253). The study was conducted from April to July 2023.

### 2.2. Subjects and Dietary Supplementation

Sixty male professional football players aged 18–35, regularly undergoing resistance and endurance training (minimum 5 sessions per week, at least 5 years’ training experience), were recruited according to specific inclusion criteria. Participants were required to be in good health, and exclusion criteria included musculoskeletal injuries, metabolic diseases, chronic illness, ongoing medication, or tobacco use. To ensure a stable baseline for microbiota analysis, individuals who had used antibiotics, medications, or probiotics within one month prior to the study were also excluded. All subjects provided written informed consent.

**Figure 1:**
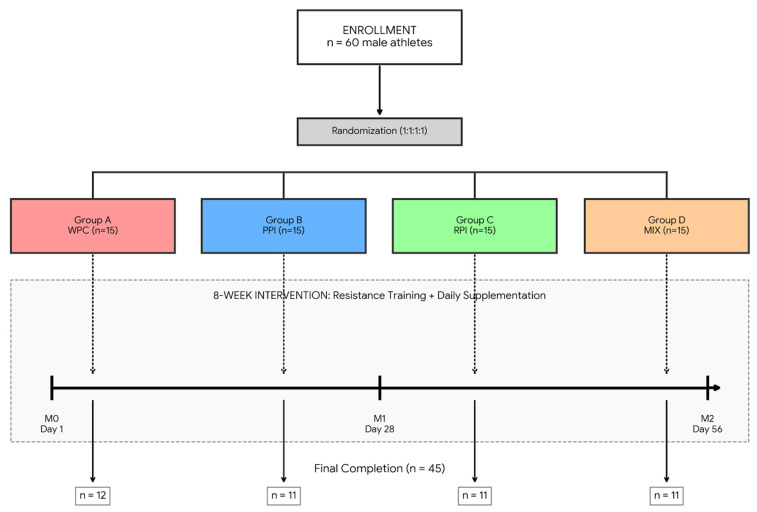
Integrated study design and participant flow diagram. The diagram illustrates the recruitment, randomization, and longitudinal timeline of the 8-week intervention. Initially, 60 elite male football players were enrolled and randomly assigned (1:1:1:1) to four protein supplementation groups: Whey Protein Concentrate (WPC), Pea Protein Isolate (PPI), Rice Protein Isolate (RPI), and a 1:1 blend of rice and pea proteins (MIX). The experimental protocol combined daily protein supplementation (30 g/day) with a standardized resistance training program. Gut microbiota assessments and dietary monitoring were performed at three distinct time points: M0 (baseline/day 1), M1 (mid-intervention/day 28), and M2 (post-intervention/day 56). The final per-protocol analysis included 45 participants who completed the full 56-day duration (WPC: *n* = 12; PPI: *n* = 11; RPI: *n* = 11; MIX: *n* = 11).

After baseline assessments, each subject was randomly assigned to one of four protein supplement groups:

- Group 1 (WPC): Whey protein concentrate
- Group 2 (RPI): Rice protein isolate
- Group 3 (PPI): Pea protein isolate
- Group 4 (MIX): Blend of rice and pea isolates (1:1 ratio)
- Participants were provided with 900 g containers of their assigned supplement, which included a buffer amount exceeding the 840 g required for each 28-day period to ensure availability. The supplements (30 g per serving, providing approximately 24 g of protein) were dissolved in 200 mL of water. Consumption occurred once daily: immediately post-training on training days or standardized before breakfast on non-training days to ensure metabolic consistency. Detailed nutrient characteristics of each supplement are presented in Table 1.
- Adherence was strictly monitored via gravimetric assessment; participants returned their containers at each follow-up visit (Day 28 and Day 56), and the residual powder was weighed to verify the 30 g daily intake. Throughout the 8-week intervention, participants were strictly instructed to avoid introducing any new dietary supplements, including probiotics or prebiotics. Habitual supplement use established prior to the study was recorded at baseline and monitored via three-day food diaries and follow-up interviews at Day 28 and Day 56. Participants who failed to demonstrate systematic supplementation or training adherence were excluded from the final per-protocol analysis to maintain the integrity of the results. Detailed reasons for participant attrition are provided in Section 3.1 and the CONSORT flow diagram (Figure 1).

### 2.3. Dietary Intervention

Food intake during the intervention was monitored using a combination of a current recording method and 24 h dietary recalls. Each participant maintained a detailed food diary for three selected days (two weekdays and one weekend day) at three time intervals: the week before the experiment, after 4 weeks of intervention, and the week before study completion. To ensure the accuracy of portion sizes and minimize underreporting, all diaries were reviewed and verified by a qualified nutritionist during mandatory follow-up meetings. These records enabled the calculation of total caloric intake, macronutrient distribution, and dietary fiber consumption using DietetykPro software web version as of 2025 (DietetykPro, Wrocław, Poland) based on national food composition tables.

Standardized nutritional guidelines were provided to all participants to maintain dietary consistency. Participants were advised to eat 4–5 meals per day at regular intervals, have their last meal at least 2 h before bedtime, and abstain from snacking between meals. Consumption of water, unsweetened fruit or herbal teas, and black coffee or tea was permitted without quantitative restrictions. Crucially, participants were strictly advised to maintain their habitual dietary patterns and avoid any significant changes in caloric or fiber intake throughout the 8-week period, ensuring that observed microbial shifts could be primarily attributed to the supplementation protocol rather than confounding dietary variation. Adherence to these guidelines was monitored by reviewing food diaries at mandatory follow-up meetings.

### 2.4. Stool Collection, DNA Extraction, Sequencing, Bioinformatics

Each participant provided a fresh stool sample at follow-up visits. Samples were aliquoted, frozen, and stored at −80 °C until analysis. DNA was extracted using the PowerFecal Pro kit (Qiagen, Hilden, Germany), which includes a two-step PCR inhibitor removal system. The V3–V8 regions of the 16S rRNA gene were amplified using universal primers 337F (5′-GACTCCTACGGGAGGCWGCAG-3′) and 1391R (5′-GACGGGCGGTGTGTRCA-3′) with LongAmp Polymerase (New England Biolabs, Ipswich, MA, USA).

Obtained amplicons were used for library preparation using the Native Barcoding Kit 96 V14 (SQK-NBD114.96) according to the manufacturer’s instructions. Sequencing was performed on R10.4.1 flowcells using a GridION device (Oxford Nanopore Technologies, Oxford, UK) under the control of MinKnow v. 6.2.6. Raw data were basecalled using the Dorado algorithm (v.7.6.7) with the super-accurate (SUP) model v. 4.3.0. Demultiplexing, as well as barcode and adapter trimming, were also performed via Dorado (v.7.6.7) according to default settings.

Raw Nanopore sequencing reads were subjected to rigorous quality control prior to downstream analysis. Reads with a quality score (QS) below 10 were discarded to minimize the impact of sequencing errors, as well as reads with a length below the threshold of 80% of the expected amplicon size. Chimeric reads were identified and removed during the preprocessing stage. Taxonomic assignment was performed using the UBLAST/USEARCH algorithm (v11.0.667) against the NCBI 16S ribosomal RNA sequences database (Bacteria and Archaea). UBLAST searches were run under default parameters; mappings with an E-value over 1 × 10^−18^ were discarded, and taxonomy assignment was based on reads mapping with the highest bit-score value.

### 2.5. Statistical Analysis

A formal sample size calculation was performed based on a one-way ANOVA framework to assess the study’s power to detect differences between the four protein supplementation groups. For a total sample size of *n* = 45 and a significance level of α = 0.05, the study achieved a power of 0.80 to detect a large effect size (f = 0.51). While the final sample size (*n* = 11–12 per group) provides moderate power for detecting significant shifts, the study is primarily characterized as exploratory, focusing on a highly controlled and unique cohort of elite athletes to identify potential metabolic trends.

Raw OTU tables were processed using MicrobiomeAnalyst 2.0 (www.microbiomeanalyst.ca). Initial preprocessing included the removal of singleton features (OTUs observed only once across all samples) to reduce spurious taxa potentially arising from sequencing errors. A low count filter was subsequently applied based on prevalence, retaining only taxa present in at least 20% of samples to minimize the influence of rare and inconsistently detected features. In addition, a low-variance filter was applied to remove the bottom 10% of features with the lowest variance across samples, thereby reducing noise and enhancing detection of biologically meaningful variation. After quality control and preprocessing, sequencing depth ranged from 43,867 to 53,964 reads per sample, indicating consistent library sizes and adequate coverage for downstream diversity and compositional analyses. These preprocessing steps were performed prior to normalization and subsequent compositional and functional analyses.

Microbiota compositional analyses were performed using MicrobiomeAnalyst 2.0. Alpha-diversity (Chao1, Shannon, Simpson indices). Differential taxonomic features were identified using Linear Discriminant Analysis Effect Size (LEfSe). Statistical significance was assessed using non-parametric Kruskal–Wallis tests followed by pairwise Wilcoxon rank-sum tests where appropriate. To control for multiple comparisons in high-dimensional microbiome data, *p*-values were adjusted using the Benjamini–Hochberg false discovery rate (FDR) correction. Features with an adjusted q-value < 0.05 and an LDA score > 1.0 were considered statistically significant and biologically relevant. Functional predictions derived from 16S rRNA gene profiles were generated using the MACADAM database and further evaluated in STAMP (v2.1.3). For pairwise comparisons of relative abundances between groups at each time point (M0, M1, M2), Welch’s *t*-tests were applied within STAMP, and resulting *p*-values were adjusted for multiple comparisons using the Storey false discovery rate (FDR) method, and corresponding q-values < 0.05 were considered statistically significant. In addition to statistical significance (q < 0.05), effect size estimates based on differences in mean relative proportions and corresponding confidence intervals (DP: Welch’s inverted method for CI) were examined to assess biological relevance. Because the study included repeated measurements within the same individuals, comparisons were conducted separately for each time point and interpreted as cross-sectional contrasts rather than longitudinal mixed-effects modeling.

Dietary intake data were analyzed using the Kruskal–Wallis test (Statistica 13.1, TIBCO Software Inc., Palo Alto, CA, USA). The corresponding H statistics and effect sizes (η^2^(H)) are presented in Supplementary Table S1. Statistical significance was set at *p* < 0.05 for dietary variables, whereas for microbiome analyses and associated features, significance was determined at q-adjusted *p* < 0.05 following multiple testing correction.

## 3. Results

### 3.1. Participant Flow and Characteristics

A total of 60 elite athletes were initially enrolled and randomized into four groups (per group). Throughout the intervention, 15 participants (25%) withdrew from the study. In accordance with the ethical approval and informed consent, participants were entitled to withdraw at any stage without providing a justification. Specific reasons for attrition included: failure to attend follow-up assessment sessions (*n* = 9), admitted non-compliance with the daily supplementation protocol (*n* = 5), and one musculoskeletal injury that prevented the continuation of the required training program. To ensure the validity of the intervention results, a per-protocol analysis was performed, including only those participants (*n* = 45) who completed the full supplementation period, maintained the training regimen, and provided all required biological samples. A CONSORT-style flow diagram illustrating the recruitment, allocation, and exclusion process has been added as Figure 1.

### 3.2. Dietary Intake

Assessment of dietary intake at various stages of the study showed that the total intake of essential macronutrients remained stable in most groups. The only exception was the RPI group, where a significant reduction in energy and fat intake was observed at M1 and M2 compared to baseline (*p* < 0.05). For all other groups and nutritional parameters, no statistically significant differences were found between day 0 and day 56, either within or between the groups (Table 2).

### 3.3. Microbiota Structure Changes Depending on Supplement Used

Analysis of alpha-diversity indices (Chao1, Shannon, Simpson) in the WPC-supplemented group revealed no statistically significant differences between time points (M0, M1, M2), indicating stable species richness and evenness throughout the intervention. In contrast to the stability observed in diversity metrics, LEfSe analysis identified differentially abundant taxa across time points using an LDA threshold > 1.0 and q < 0.05. *Faecalibacterium* and *Blautia* remained the dominant genera at all sampling points. Genera *Enterocloster* and *Desulfohalotomaculum* were significantly enriched at baseline (M0), whereas genera *Sutterella*, *Hungatella*, and *Anaerosporobacter* showed significant enrichment following supplementation (M1/M2).

**Figure 2:**
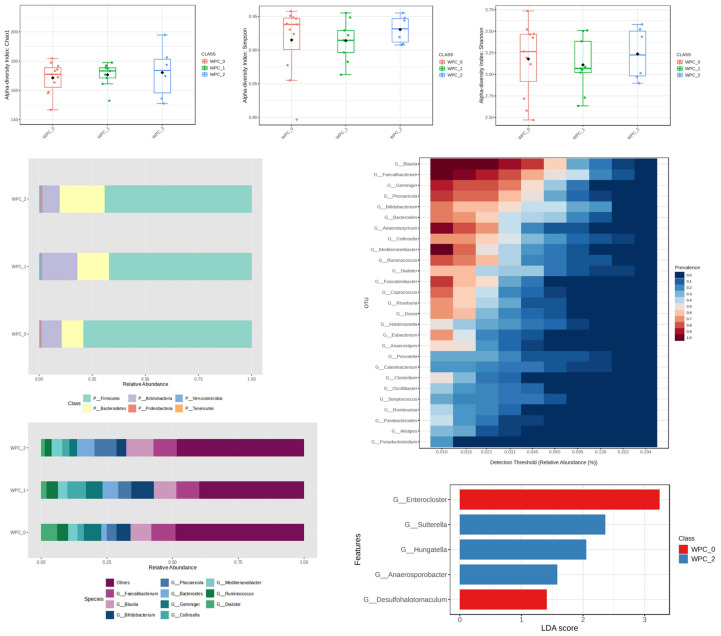
Changes in bacterial microbiota structure for WPC supplementation depending on the time of the experiment.

At the phylum level, descriptive analysis suggested a transient increase in *Actinobacteria* and *Bacteroidetes* at week 4, followed by a sustained presence of *Bacteroidetes* at week 8 (Figure 2). However, these broader compositional patterns were primarily driven by the genus-level differences identified in the LEfSe analysis rather than by statistically significant shifts at higher taxonomic ranks (Figure 2).

**Figure 3:**
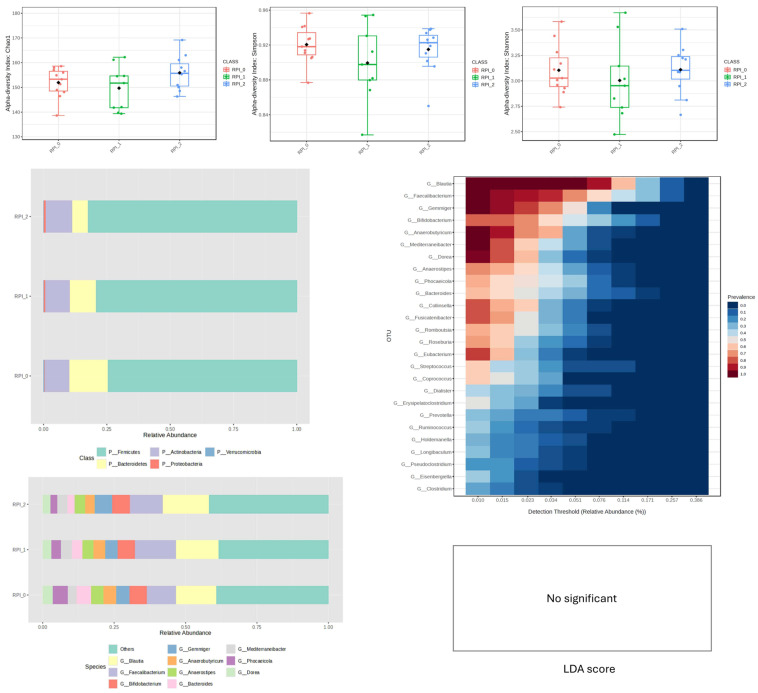
Changes in bacterial microbiota structure for RPI supplementation depending on the time of the experiment.

In the RPI-supplemented group, alpha-diversity indices did not change significantly over time compared to baseline. At the taxonomic level, substantial inter-individual variability was observed. Visual evaluation of relative abundance plots (Figure 3) suggested a possible increase in *Firmicutes* and a numerical decrease in *Bacteroidetes* at weeks 4 and 8, accompanied by a modest increase in *Proteobacteria*. However, despite these observable differences in mean relative abundance, LEfSe analysis did not identify any statistically significant differentially abundant taxa (LDA score > 2.0, q < 0.05) between time points.

These findings indicate that although RPI supplementation may have been associated with individual-level variability, it did not result in consistent or statistically significant restructuring of the gut microbiome within the cohort (Figure 3).

**Figure 4:**
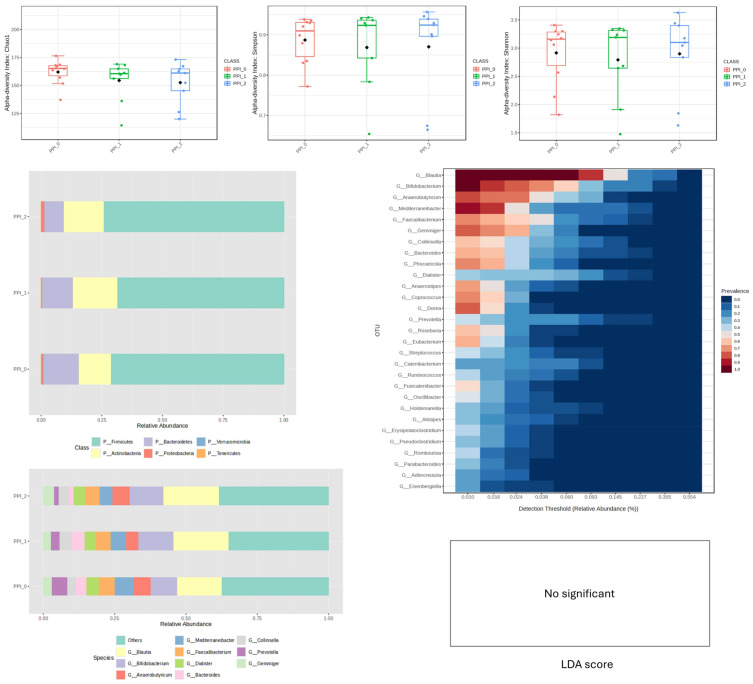
Changes in bacterial microbiota structure for PPI supplementation depending on the time of the experiment.

Similarly, PPI supplementation was not associated with significant changes in alpha-diversity indices compared to baseline. Substantial intra-group variability was observed at the taxonomic level, indicating heterogeneous individual responses. Descriptive analysis of mean relative abundance data suggested a possible reduction in *Bacteroidetes* and a numerical increase in *Proteobacteria* and *Bifidobacterium*, particularly at week 4 (Figure 4). However, these compositional patterns did not reach statistical significance after correction for multiple comparisons in the LEfSe analysis (q > 0.05).

The core microbiome, predominantly composed of genera *Blautia*, *Bifidobacterium*, and *Mediterraneibacter*, remained relatively stable throughout the intervention. Accordingly, no specific taxonomic biomarkers were significantly associated with PPI supplementation in this cohort (Figure 4).

**Figure 5:**
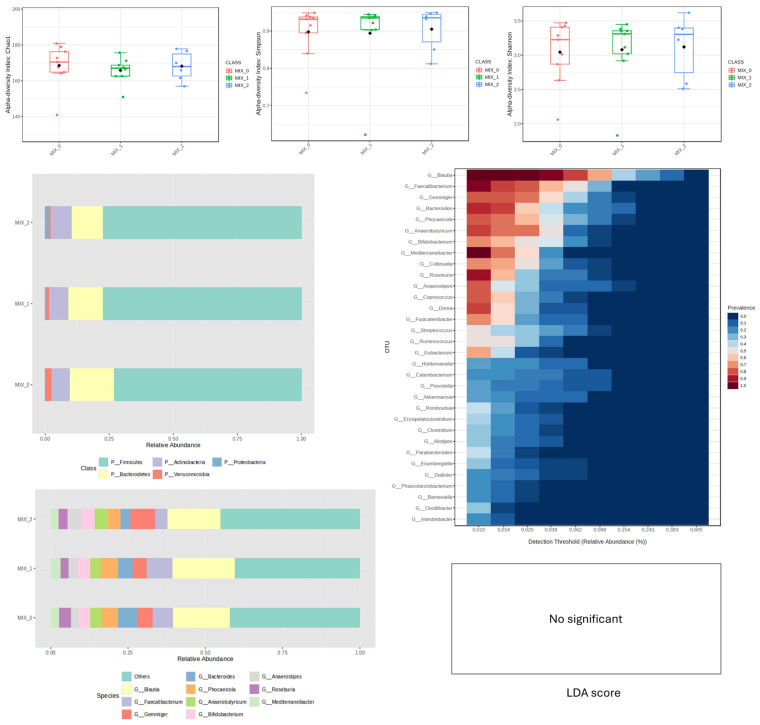
Changes in bacterial microbiota structure for MIX supplementation depending on the time of the experiment.

In the MIX group, alpha diversity indices remained stable at both 4 and 8 weeks compared to baseline. Considerable inter-individual dispersion was observed, indicating heterogeneous responses to supplementation. Visual evaluation of relative abundance plots (Figure 5) suggested a possible increase in *Firmicutes* and *Gemmiger*, accompanied by a numerical decrease in *Bacteroidetes* and *Faecalibacterium* over time. However, consistent with the RPI and PPI groups, these observed compositional patterns did not reach statistical significance in the LEfSe analysis (q > 0.05).

Therefore, under the tested conditions, mixed protein supplementation was not associated with statistically significant alterations in the overall taxonomic structure of the gut microbiome (Figure 5).

### 3.4. Comparative Analysis of Dietary Variants

**Figure 6:**
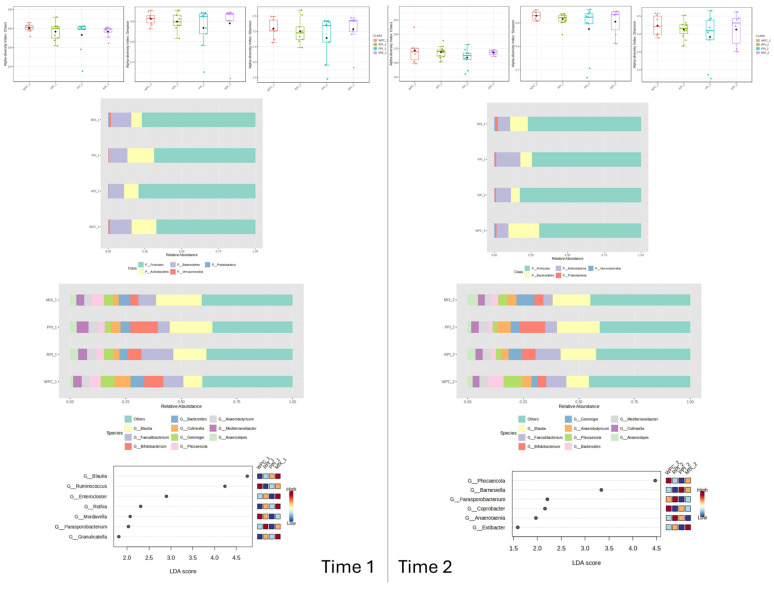
Comparison of the effect of supplements on gut microbiota structure after 28 and 56 days of supplementation.

*Firmicutes* dominated the bacterial community regardless of intervention length, and no statistically significant differences in phylum-level relative abundance were detected between supplementation groups. Nevertheless, descriptive analysis of relative abundance plots suggested temporal patterns (Figure 6). After 4 weeks, WPC and PPI variants showed numerically higher *Actinobacteria* abundance compared to MIX and RPI, whereas RPI was characterized by the highest relative abundance of *Firmicutes* and lower proportions of *Bacteroidetes* and *Actinobacteria* (<10%). WPC and MIX also exhibited a slight increase in *Verrucomicrobia* (~1%).

After 8 weeks, WPC displayed a numerically higher proportion of *Bacteroidetes* (~20%) and lower *Firmicutes* (~70%). PPI and RPI did not exceed 10% *Bacteroidetes*, while PPI showed relatively higher *Actinobacteria* abundance (~45%). A modest increase in *Proteobacteria* was observed across all supplementation types, with MIX reaching approximately 3%.

At the genus level, temporal variability was also observed (Figure 6). After 4 weeks, *Blautia* was numerically more abundant (~20%) in MIX and PPI and less represented in WPC (~8%). RPI was distinguished by a high relative abundance of *Faecalibacterium* (~50%) and moderate Bifidobacterium levels. These genus-level differences, however, did not reach statistical significance and should be interpreted as descriptive trends (Figure 6).

### 3.5. Changes in Abundance Functional Group of Bacteria

**Figure 7:**
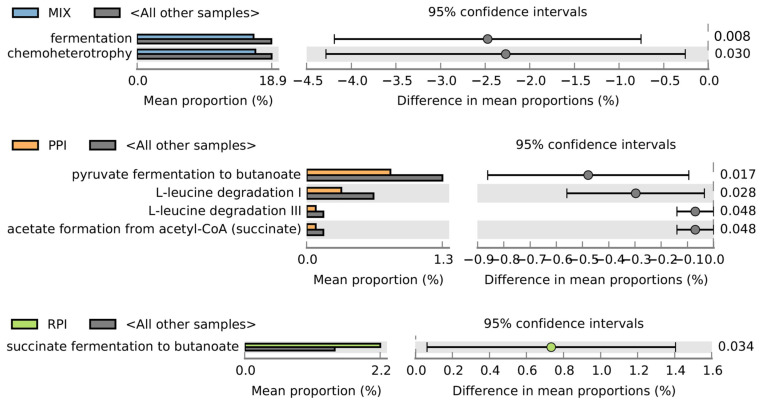
Observed predicted metabolic interactions after 28 days of supplementation.

Functional inference analysis revealed subtle shifts in the predicted metabolic potential of the microbiota. After 4 weeks, the MIX group showed a decrease in the predicted relative abundance of sequences associated with fermentation and chemoheterotrophy, which may suggest a shift in the community’s metabolic potential. In the PPI group at week 4, a lower predicted abundance of genes linked to pyruvate fermentation to butanoate and acetate formation was observed, alongside a reduction in sequences associated with L-leucine degradation. In contrast, RPI supplementation at week 4 coincided with an increased predicted proportion of sequences related to succinate fermentation to butanoate, potentially contributing to the community’s capacity for SCFA biosynthesis (Figure 7).

**Figure 8:**
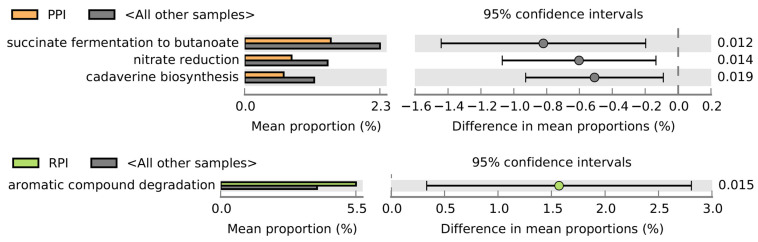
Observed predicted metabolic interactions after 56 days of supplementation.

After 8 weeks, PPI supplementation was associated with a lower predicted abundance of taxa involved in succinate fermentation to butanoate, cadaverine biosynthesis, and nitrate reduction. Similarly, the RPI group at 8 weeks showed a higher predicted potential for aromatic compound degradation (Figure 8). These findings represent inferred functional capacities based on 16S rRNA gene amplicon data and should be interpreted as predictive rather than direct measurements of metabolic activity.

## 4. Discussion

The findings of this study indicate that supplementation with proteins of various origins (MIX, WPC, RPI, PPI) did not lead to significant alterations in the overall diversity of the gut microbiota, as confirmed by stable alpha-diversity indices. Nevertheless, detailed taxonomic and functional analyses revealed subtle but potentially relevant shifts in the predicted metabolic potential of the microbiome [8,9].

WPC supplementation demonstrated stability in the Chao1, Shannon, and Simpson indices and progressive stabilization of the microbial ecosystem. At the phylum level, a significant increase in *Actinobacteria* and *Bacteroidetes* was observed after 4 weeks, with *Actinobacteria* returning to baseline and *Bacteroidetes* rising further at 8 weeks. The increased abundance of *Bacteroidetes* after 8 weeks may indicate a shift toward taxa previously associated with SCFA-related metabolic pathways; however, SCFA concentrations were not directly measured. Importantly, recent research by Govindan et al. provides direct in vivo support for these functional inferences. In their 12-week resistance training trial, participants supplementing with plant protein (lentil) exhibited significantly higher fecal concentrations of butyrate and acetate—measured via gas chromatography—compared to those consuming animal protein (whey/egg). This suggests that the predicted increase in fermentation pathways observed in our plant-protein groups (RPI/PPI) likely translates into tangible metabolic shifts [10]. This is consistent with in vitro studies on whey protein hydrolysate and animal studies demonstrating increased short-chain fatty acid production and the restoration of key bacterial families following protein intervention [11,12]. Genera *Blautia* and *Faecalibacterium* dominated at the genus level, while fluctuations were noted for *Bifidobacterium*, *Bacteroides*, *Collinsella*, *Phocaeicola*, and *Gemmiger*. LEfSe analysis revealed significant differences between WPC0 and WPC2 groups, particularly for genera *Enterocloster*, *Desulfohalotomaculum*, *Suterella*, *Huangatella*, and *Anaerosporobacter*, indicating functionally meaningful ecological shifts [13,14].

The RPI group demonstrated high taxonomic stability, but after 4 weeks, there was an increase in taxa classified as succinate-to-malate fermenters, which may suggest a shift in predicted SCFA-related metabolic potential; nevertheless, no direct metabolite data were obtained [15]. Eight weeks of supplementation increased bacteria involved in aromatic compound degradation, which may be beneficial for immunoregulation. A reduction in these bacteria may diminish production of protective metabolites and increase risk for inflammatory and autoimmune conditions [16,17].

PPI supplementation did not result in significant changes in alpha-diversity, but samples showed marked intra-group variability. After 4 weeks, there was an increase in Bifidobacterium (above 20%) and a reduction in *Bacteroides* and *Prevotella*. There was also a reduction in pyruvate-to-butyrate fermenters, acetate-producing bacteria, and leucine-degraders. These changes may reflect alterations in taxa associated with SCFA-related pathways; however, direct quantification of SCFAs or other metabolites was not performed [9]. After 8 weeks, there was a further decrease in succinate-to-malate fermenters and nitrate-reducing bacteria, which could alter N-NO_3_ levels and contribute to inflammatory disorders.

The role of bacteria potentially related to cadaverine biosynthesis requires further clinical study, as their significance in the gut environment is still not fully understood [18].

MIX and PPI supplementation was associated with dynamic shifts in the microbiome composition, including reductions in taxa predicted to contribute to malate and acetate production. The observed increase in Bifidobacterium following PPI supplementation aligns with previous studies on pea protein [19], which reported similar effects in multi-ingredient interventions and underscores the complexity of microbial interactions. Both PPI and RPI showed a decline in *Bacteroidetes*, contrasting with findings from animal models that indicated the opposite trend [20] however, results from human studies regarding both Bacteroides and Bifidobacterium are consistent with our findings [21]. MIX supplementation did not alter alpha-diversity but revealed substantial inter-individual variability, including *Firmicutes* dominance at 4 weeks, a corresponding decrease in *Bacteroidetes*, and reduced *Verrucomicrobia*. The increase in *Gemmiger* alongside a decline in *Faecalibacterium*, as well as reductions in predicted fermentative and heterotrophic taxa, points to shifts in the predicted metabolic potential of the microbiota, potentially affecting SCFA and other metabolite-associated pathways.

Interpreting these results—particularly the natural production of proteases by damaged legume plant cells (PPI variant)—protein fractions as well as the presence of exogenous proteases and nitrogenous compounds appear to play a significant role in shaping the gut microenvironment, potentially promoting the growth of ammonifying and denitrifying bacteria. This shift may reflect alterations in taxa linked to butyrate-related metabolic pathways, although functional consequences cannot be confirmed without targeted metabolomic analysis. A similar protease-driven mechanism has been described in the context of legume-based feeds for livestock, but direct parallels with human gut microbiota remain speculative in the absence of corresponding metabolite data [22].

Accordingly, the present findings should be regarded as hypothesis-generating. Interpretation of these findings must remain cautious. While dietary protein composition—including plant-derived protein fractions—may theoretically influence nitrogen availability and microbial metabolism, the present study design does not allow mechanistic conclusions. Functional predictions derived from 16S-based taxonomic profiles provide only an estimation of potential metabolic capacity and cannot substitute for targeted metagenomic or metabolomic analyses.

Bel Lassan et al. [23] investigated the effects of high-protein milk-based supplementation during moderate energy restriction in individuals with overweight or obesity and metabolic syndrome in a 12-week randomized, double-blind trial. Compared with an isocaloric preparation with lower protein content, the high-protein intervention led to greater reductions in visceral and total adipose tissue, better preservation of lean body mass, and decreased circulating inflammatory markers (CRP, TNFα), without significant between-group differences in classical cardiometabolic parameters. Metagenomic profiling of the gut microbiota revealed minimal alterations in diversity and taxonomic composition. Nevertheless, in the high-protein group, a pronounced activation of functional modules related to amino acid metabolism—particularly biosynthetic pathways—was observed, with the magnitude of this response correlating with dietary protein intake rather than the extent of weight loss. Furthermore, an increase in microbiome gene diversity among participants with initially low richness was associated with greater reductions in body weight and fat mass, irrespective of the supplement type. These findings indicate that the beneficial metabolic effects of dietary intervention are primarily mediated through modulation of microbial metabolic functions rather than through profound taxonomic restructuring of the gut microbiota. Similarly, in our study, we also observed only moderate rearrangements in the gut bacterial microbiota composition, accompanied, however, by pronounced shifts in the predicted functional metabolic potential, particularly within pathways related to amino acid metabolism. This convergence with the findings of Bel Lassan et al. [23] reinforces the concept that dietary protein intake may primarily modulate the functional capacity of the gut microbiota rather than induce extensive taxonomic restructuring.

### Limitations and Generalizability

This study has several limitations that should be considered when interpreting the results. First, regarding statistical analysis, we employed cross-sectional comparisons at specific time points rather than longitudinal mixed-effects modeling. While this approach allows for the identification of specific differences at days 28 and 56, it limits the ability to fully infer continuous longitudinal evolution and subject-specific random effects over time. Future studies should employ mixed-effects models on larger cohorts to robustly capture these longitudinal dynamics.

Second, the functional analyses presented here are based on predictive metagenomics inferred from 16S rRNA gene amplicon data (MACADAM/STAMP). While this approach provides valuable insights into the metabolic potential of the microbial community, it does not confirm the actual production of metabolites (e.g., SCFAs, polyamines). Therefore, terms such as “biosynthesis” or “degradation” in our results refer to the abundance of genes annotated to these pathways, not the measured metabolic flux. Future studies should incorporate targeted metabolomics to validate these predictions.

Regarding generalizability, the study cohort consisted exclusively of young male professional football players. Consequently, the observed shifts in microbiota composition and predicted metabolic functions may not directly translate to female athletes, endurance-based populations, or recreational users due to differences in hormonal profiles and training metabolic demands.

However, studying elite cohorts is justified by the fact that professional sports nutrition often sets the standard for amateur and recreational populations. As recreational athletes increasingly adopt professional supplementation protocols to enhance performance, understanding the impact of these products in a highly controlled elite setting is of high practical relevance. Nonetheless, broader nutritional recommendations based on these results should be applied with caution to the general population.

## 5. Conclusions

In summary, although protein supplementation affects only a part of the microbiota, even small taxonomic or functional changes may contribute to shifts in the predicted metabolic potential of the gut microbial community. However, the physiological or health-related implications of these changes remain to be confirmed by targeted metabolomic and clinical assessments. The observed differences in the predicted abundance of genes associated with SCFA production and fermentation pathways suggest differential effects of supplements on inferred metabolic potential; however, further studies on direct assessment of fermentative activity or metabolite concentrations are required. Further research should focus on functional, metabolomic, and immunological analyses to better understand the consequences of these changes for athletes and to develop individualized supplementation plans.

## Figures and Tables

**Table 1:** Composition of protein powders.

Measure	WPC	RPI	PPI	MIX
Protein [%]	80	83.5	83.4	83.5
Carbohydrates [%]	5.5	<1	<1	<1
of which sugars [%]	5.5	0	0	0
Dietary fibre [%]	0	4.6	4.1	4.4
Fat [%]	5.9	4.3	2.0	3.2

WPC: whey protein concentrate, RPI: rice protein isolate, PPI: pea protein isolate, MIX: a combination of pea protein isolate and rice protein isolate in a 1:1 ratio.

**Table 2:** Nutrient intake per day without nutritional supplementation.

Diet Intake		Energy (kcal)	Protein (g)	Fat (g)	Carbohydrates (g)	of Which Sugars (g)	Fiber (g)
WPC	M0	2612 ± 582	131.4 ± 28.1	102.8 ± 29.3	293.3 ± 84.1	75.8 ± 42	16.9 ± 8.7
	M1	2291 ± 353	129.6 ± 28.1	75.7 ± 25.9	280.5 ± 64.7	67.2 ± 30.7	21.1 ± 7.1
	M2	2379 ± 431	125.1 ± 25.6	83.2 ± 29.2	290.6 ± 65.8	81.5 ± 49.5	19.5 ± 4.0
RPI	M0	2585 ± 369 ^a^	135 ± 35.3	93.5 ± 26.9 ^a^	305.5 ± 63.6	73.9 ± 42.3	21.3 ± 9.4
	M1	2286 ± 330 ^b^	128.8 ± 32.6	72.3 ± 21.8 ^b^	284 ± 59.5	101.5 ± 83.3	24.1 ± 11.1
	M2	2198 ± 262 ^b^	119.6 ± 23.3	71.6 ± 19.7 ^b^	271.7 ± 61.7	95.7 ± 85.5	23.5 ± 11.1
PPI	M0	3323 ± 818	170.2 ± 54.8	107.9 ± 36.7	417.2 ± 119.8	127.4 ± 33.6	21.9 ± 6.8
	M1	2888 ± 449	154.3 ± 29.3	96.4 ± 30.4	351.6 ± 61.7	103.6 ± 16.0	23.8 ± 6.2
	M2	2922 ± 408	160.5 ± 30.4	98.7 ± 28.4	349.1 ± 65	100.65 ± 14.5	24.1 ± 6.2
MIX	M0	2587 ± 513	126.9 ± 9	89.9 ± 23.4	321.3 ± 88.3	81.7 ± 43.3	20.3 ± 7.0
	M1	2362 ± 613	133.8 ± 41.2	87 ± 25.7	264.7 ± 67.4	68.9 ± 34.4	16.3 ± 4.8
	M2	2431 ± 652	130.2 ± 40.8	98.9 ± 30.1	259.6 ± 63.5	77.6 ± 42.1	15.7 ± 2.7

M0: initial time, M1: after 28 days of supplement consumption, M2: after 56 days of supplement consumption. WPC: whey protein concentrate, RPI: rice protein isolate, PPI: pea protein isolate, MIX: a combination of pea protein isolate and rice protein isolate in a 1:1 ratio. Values are means ± standard deviation. Superscripts (a, b) denote statistically significant differences between time points within the same group (post hoc comparisons, *p* < 0.05).

## Data Availability

The raw sequencing data (V3–V8 16S rRNA) presented in this study are openly available in the National Center for Biotechnology Information (NCBI) Sequence Read Archive (SRA) under BioProject accession number PRJNA1144017.

## Supplementary Materials

The following supporting information can be downloaded at: https://www.mdpi.com/article/10.3390/nu18050768/s1, Table S1: Kruskal–Wallis H statistics and corresponding effect sizes (η^2^(H)) for between-group comparisons.
